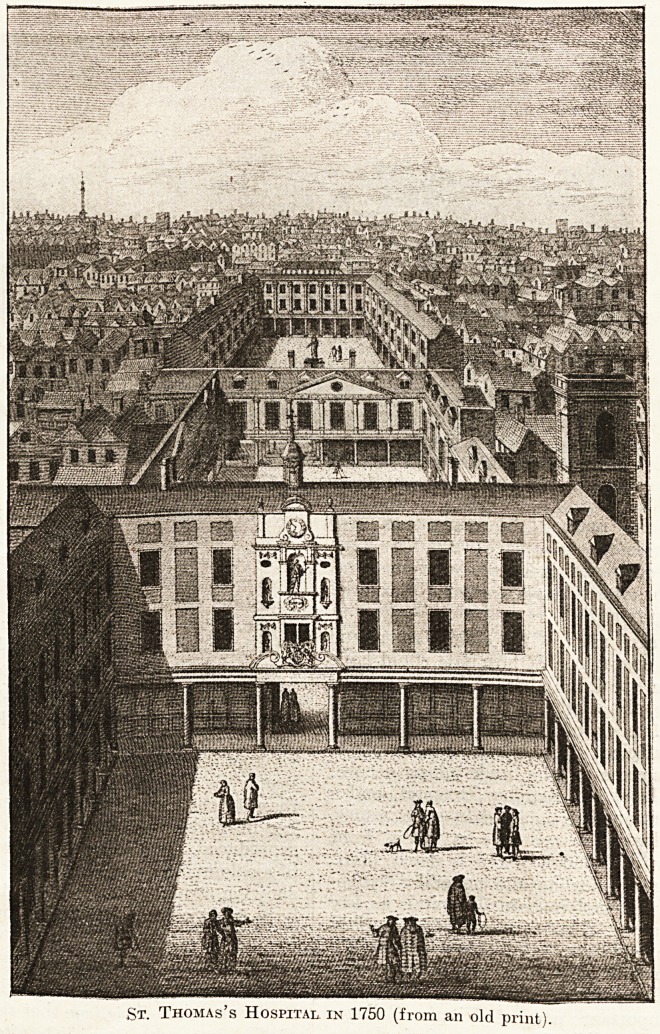# As Depicted by Engraved Views.—I

**Published:** 1920-11-13

**Authors:** 


### THE HISTORY OF HOSPITALS

### AS DEPICTED BY ENGRAVED VIEWS.—I.

The eighteenth century was the period when the print sellers were in the hey-dey of their prosperity, but early in the nineteenth century changing tastes and changing conditions led to other methods of art as applied to engraved illustrations of people, views, historical and fancy scenes, and various subjects. The change was probably brought about chiefly by the introduction of the steel plate and the resulting large editions taking the place of the quickly - worn copper plate and the small output of impressions. The comparative cheapness of the system of producing a large number of pulls from a plate naturally led to the production of books of engravings, and pushed out of the market the engravings sold singly. Thus it is that we have very few large and rare engraved views of hospitals, as most of these institutions were founded after the era of copper-plate engraving.

St. Bartholomew's Hospital in 1723 (from an old print).

The publishers of most of the older, larger, and more important engravings of hospitals could hope to attract but a small number of buyers. The plates were wrought singly and in sets, after the governors and friends of the institution had subscribed for a sufficient number of copies to make the financial success of the undertaking secure. The Foundling Hospital, Bethlem Hospital, and a few other institutions are depicted in such issues, but the first illustration we give is taken from "Stow's Survey of London," and is dated 1723. It is interesting as showing the general arrangement of St. Bartholomew's Hospital a few years before James Gibbs commenced in 1729 to erect the fine buildings, not finished until 1770, which now surround the great quadrangle. The fine work of Gibbs is worthy of the man who designed an important part of King's College, Cambridge, the Radcliffe Library at Oxford, the Church of St. Martin's-in-the-Fields, and other London churches. Of prints of St. Bartholomew's subsequent to this important alteration there are a great number, and Mr. Arthur Watkins, the late Steward, possessed as nice a private collection of them as the writer has seen.

### Two Hundred Years Ago.

Under the engraving here reproduced there is this quaint and informative inscription:

This Hospital is erected for poor Sick, Wounded and Diseased Persons, where great care is taken of them, and all Necessaries for Food, Lodging, Attendance, Physick and proper Medicaments for their Cure are Administered to them. It formerly belong'd to ye Priory of St. Bartholomew in West Smithfield, but coming into Henry 8th's hands at ye suppression, was by him founded afresh. It was afterwards encouraged by Edwd. y 6th and since then by many other Benefactors; so that ye buildings have lately been repair'd and greatly enlarg'd, and ye number of ye reliev'd infirm Poor much increas'd. By the report of this Hospital made in this present year 1723, it appears that in the year last past 3381 persons were cured and discharged ye Hospital; many of which were reliev'd with Money and other Necessaries at their departure, 217 were buried after much charge in their Illness, & 565 are still remaining under Cure. This Hospital is under ye care of ye Lord Mayor &c. of London.

The church of St. Bartholomew-the-Less and the gateway with the statue of Henry VIII. remain, practically all the rest has disappeared. The row of houses, backing on the hospital precincts, each with its booth, remind us that Smithfield market came nearer St. Bartholomew's two hundred years ago than it does to-day. So, by means of a large number of plates, commencing with the one we have reproduced, and terminating with a reproduction of a coloured drawing by Horace von Ruith in 1916, showing wounded soldiers in the quadrangle, we have fairly complete material for the illustration of the history of our oldest hospital.

### St. Thomas's Before the Great Move.

Another hospital with a good pictorial history is St. Thomas's. It is true that there is no early view of the time when in 1553, after long disuse, the building was ordered to be "repayred and maid sweete and ready to receive 300 poore." But an idea of what the hospital was like in the seventeenth century may be obtained from a print in Stow's "Survey," with the following descriptive matter:

**The History of Hospitals**—(*continued*).

This Hospital was first founded in 1213 & named an Almery for Converts and poor Children; in 1215 it was converted into a priory for Canons Regular and at ye Reformation was surrendered to ye Crown. King Edwd. 6th gave it in a very decay'd Condition to ye Citizens of London, who immediately repair'd it for Poor, Impotent, Lame, and Diseas'd Persons, but it has lately been entirely new built, and has received sev'l Enlargements and additional Buildings, by wch it is made capable of receiving a far greater Number of Patients than ever, since ye Foundation. The prodigious relief this Hospital affords to miserable Objects (many of whom wou'd otherwise perish) will appear from ye Numb'rs cured there, wch were 4721 during ye Year last past, and sevl of them were reliev'd with Money and other Necessaries at their departure: 353 were Buried thence after much charge in their illness, and 621 are still remain'g under Cure. The L'd May'r, Alderman, and 250 Others chiefly Citizens and Merchants, are Governors hereof.

St. Thomas's Hospital in 1750 (from an old print).

Another and larger engraving, probably produced about 1750, shows that another storey has been added to the first of the three quadrangles, or rather the top floor with dormer windows has been enlarged and squared, and there is a statue of Edward VI. over the gateway between the first and second quadrangles, which is now to be seen at the present building opposite the Houses of Parliament.

The Boy King's Statue.

We have before us as we write a print showing the Boy King's statue standing in a railed-in space in the second quadrangle of the old building. Possibly it is the same in another position. The cupola above the statues and clock enclosed a bell used for sundry purposes, including the summoning of patients and staff to meals.

Some Forgotten Statues.

There were also four other smaller stone figures, shown in the print, on the same wall. These Mr. G. Q. Roberts, the present Secretary of the hospital, unearthed from a cellar in quite recent years, the statues evidently having come with the other goods and chattels when the great removal took place. It is not generally known that one of the eighteenth-century wings of the old hospital still stands in St. Thomas's Street, and is used as a post-office, and that part of the site has not yet been built on. There is, we believe, a possibility that Guy's Hospital may extend in this direction.

In the furthermost quadrangle will be seen the statue of Lord Mayor Sir R. Clayton, one of the best friends of St. Thomas's, who was President when the hospital was rebuilt in 1693-95. It is probable that the engraving in Stow's "Survey," although appearing much later than the first date, is from an out-of-date drawing, and that the print we reproduce represents the hospital as it was during practically the whole of the eighteenth century.

St. Thomas's Hospital is described in the letterpress of one of Ackerman's early nineteenth century books of coloured aquatints as "for the sick and lame, especially sailors."

## Figures and Tables

**Figure f1:**
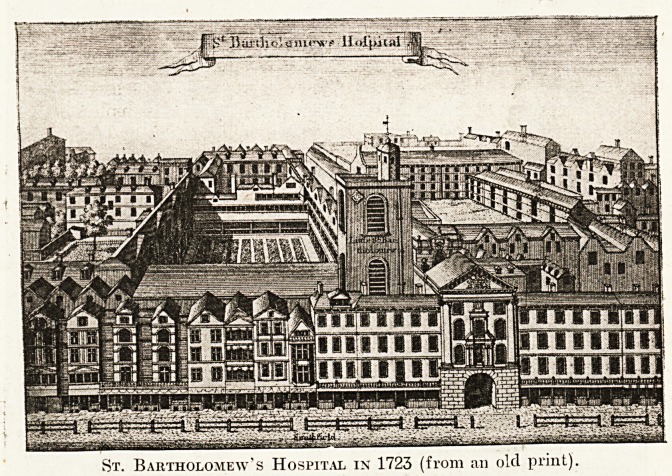


**Figure f2:**